# 5-(Anthracen-9-yl)-3-(4-nitro­phen­yl)-1-phenyl-4,5-dihydro-1*H*-pyrazole

**DOI:** 10.1107/S1600536810038912

**Published:** 2010-10-09

**Authors:** Bao-Li Dong, Ming-Liang Wang, Yong-Hua Li

**Affiliations:** a School of Chemistry and Chemical Engineering, Southeast University, Nanjing 211189, People’s Republic of China

## Abstract

In the title compound, C_29_H_21_N_3_O_2_, the five-membered pyrazoline ring is nearly planar, the maximum deviation being 0.037 (3) Å. The anthracene ring system is approximately perpendicular to the central pyrazoline ring, making a dihedral angle of 86.55 (16)°, whereas the two attached benzene rings are oriented at smaller dihedral angles of 12.9 (2) and 14.7 (2)°with respect to the pyrazoline ring. An intra­molecular C—H⋯N hydrogen bond is observed.

## Related literature

For applications of pyrazoline derivatives, see: Shaharyar *et al.* (2006 ▶); Christoph *et al.* (2003 ▶); Parmar *et al.* (1974 ▶); Prasad *et al.* (2005 ▶). For a related pyrazoline compound, see: Krishna *et al.* (1999 ▶).
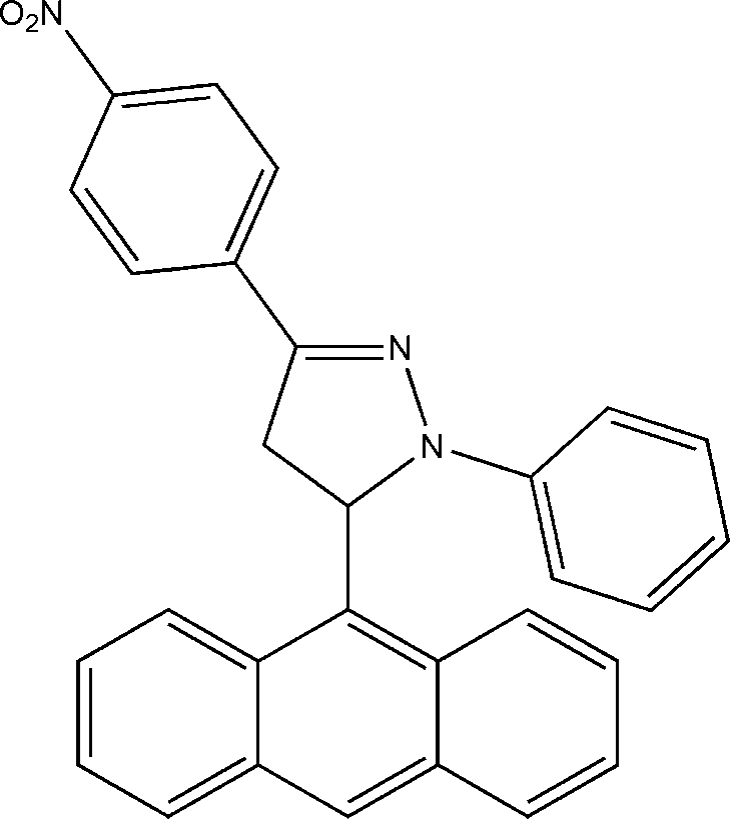


## Experimental

### 

#### Crystal data

C_29_H_21_N_3_O_2_
*M* *_r_* = 443.49
Orthorhombic, 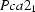

*a* = 23.023 (5) Å
*b* = 10.195 (2) Å
*c* = 9.2005 (18) Å
*V* = 2159.6 (7) Å^3^
*Z* = 4
Mo *K*α radiation
μ = 0.09 mm^−1^
*T* = 293 K
0.20 × 0.20 × 0.20 mm

#### Data collection

Rigaku Mercury2 diffractometer
17812 measured reflections
2023 independent reflections
1675 reflections with *I* > 2σ(*I*)
*R*_int_ = 0.068

#### Refinement

*R*[*F* ^2^ > 2σ(*F* ^2^)] = 0.044
*wR*(*F* ^2^) = 0.095
*S* = 1.08
2023 reflections
308 parameters
2 restraints
H-atom parameters constrained
Δρ_max_ = 0.14 e Å^−3^
Δρ_min_ = −0.16 e Å^−3^

### 

Data collection: *CrystalClear* (Rigaku, 2005 ▶); cell refinement: *CrystalClear*; data reduction: *CrystalClear*; program(s) used to solve structure: *SHELXTL* (Sheldrick, 2008 ▶); program(s) used to refine structure: *SHELXTL*; molecular graphics: *SHELXTL*; software used to prepare material for publication: *SHELXTL* .

## Supplementary Material

Crystal structure: contains datablocks I, global. DOI: 10.1107/S1600536810038912/xu5037sup1.cif

Structure factors: contains datablocks I. DOI: 10.1107/S1600536810038912/xu5037Isup2.hkl

Additional supplementary materials:  crystallographic information; 3D view; checkCIF report

## Figures and Tables

**Table 1:** Hydrogen-bond geometry (Å, °)

*D*—H⋯*A*	*D*—H	H⋯*A*	*D*⋯*A*	*D*—H⋯*A*
C1—H1*A*⋯N2	0.93	2.59	3.422 (5)	150

**Fig. 1.**
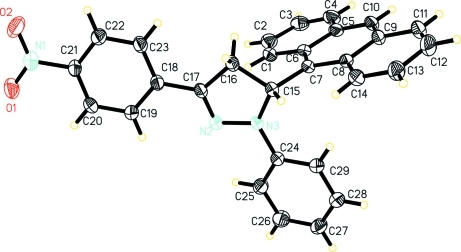
Perspective structure of the title compound, showing the atom-numbering scheme. Displacement ellipsoids are drawn at the 30% probability level.

## supplementary crystallographic information

### Comment

The derivatives of pyrazoline are mostly used in medicine, such as antibacterial (Shaharyar *et al.*, 2006), antidepressant (Prasad *et al.*, 2005) and anticonvulsant (Parmar *et al.*, 1974), furthermore they also have application in cell biological study due to their simple structure and favorable photophysical properties (Christoph *et al.*, 2003). Here we report the structure of the title compound (I), a new derivative of pyrazoline.

In the pyrazoline ring, the bond length of C17=N2 [1.287 (4) Å] indicates a to the normal C=N bond (1.28 Å), while the N2—N3 distance [1.366 (4) Å] agrees with the expected values (Krishna *et al.*, 1999). The mean plane of anthryl ring makes dihedral angles of 84.98 (9) and 82.81 (8)°, with the benzene ring and 4-nitrophenyl group, respectively.

### Experimental

3-(9-Anthryl)-1-(4-nitrophenylprop)-2-en-1-one (3 mmol, 1.0 g) and phenylhydrazine (6.5 mmol, 0.7 g) were dissolved in 10 ml acetic acid. The mixture was stirred for 8 h at refluxing temperature to give red solid. The product was isolated and recrystallized from ethanol/ethyl acetate (1:1 *v*/*v*) mixed solution, red single-crystal of (1) was obtained.

### Refinement

H atoms were positioned geometrically and refined using a riding model, with C—H = 0.93–0.97 Å and U_iso_(H) = 1.2U_eq_(C). As no significant anomalous scatterings, Friedel pairs were merged.

### Crystal data

**Table:**

C_29_H_21_N_3_O_2_	*F*(000) = 928
*M**_r_* = 443.49	*D*_x_ = 1.364 Mg m^−^^3^
Orthorhombic, *P**c**a*2_1_	Mo *K*α radiation, λ = 0.71073 Å
Hall symbol: P 2c -2ac	Cell parameters from 2023 reflections
*a* = 23.023 (5) Å	θ = 2.6–25.0°
*b* = 10.195 (2) Å	µ = 0.09 mm^−^^1^
*c* = 9.2005 (18) Å	*T* = 293 K
*V* = 2159.6 (7) Å^3^	Prism, red
*Z* = 4	0.20 × 0.20 × 0.20 mm

### Data collection

**Table:**

Rigaku Mercury2 diffractometer	1675 reflections with *I* > 2σ(*I*)
Radiation source: fine-focus sealed tube	*R*_int_ = 0.068
graphite	θ_max_ = 25.0°, θ_min_ = 3.1°
Detector resolution: 13.6612 pixels mm^-1^	*h* = −27→27
CCD_Profile_fitting scans	*k* = −12→12
17812 measured reflections	*l* = −10→10
2023 independent reflections	

### Refinement

**Table:**

Refinement on *F*^2^	Primary atom site location: structure-invariant direct methods
Least-squares matrix: full	Secondary atom site location: difference Fourier map
*R*[*F*^2^ > 2σ(*F*^2^)] = 0.044	Hydrogen site location: inferred from neighbouring sites
*wR*(*F*^2^) = 0.095	H-atom parameters constrained
*S* = 1.08	*w* = 1/[σ^2^(*F*_o_^2^) + (0.0418*P*)^2^ + 0.3731*P*] where *P* = (*F*_o_^2^ + 2*F*_c_^2^)/3
2023 reflections	(Δ/σ)_max_ < 0.001
308 parameters	Δρ_max_ = 0.14 e Å^−^^3^
2 restraints	Δρ_min_ = −0.16 e Å^−^^3^

### Special details

**Table:**

Geometry. All e.s.d.'s (except the e.s.d. in the dihedral angle between two l.s. planes) are estimated using the full covariance matrix. The cell e.s.d.'s are taken into account individually in the estimation of e.s.d.'s in distances, angles and torsion angles; correlations between e.s.d.'s in cell parameters are only used when they are defined by crystal symmetry. An approximate (isotropic) treatment of cell e.s.d.'s is used for estimating e.s.d.'s involving l.s. planes.
Refinement. Refinement of *F*^2^ against ALL reflections. The weighted *R*-factor *wR* and goodness of fit *S* are based on *F*^2^, conventional *R*-factors *R* are based on *F*, with *F* set to zero for negative *F*^2^. The threshold expression of *F*^2^ > σ(*F*^2^) is used only for calculating *R*-factors(gt) *etc*. and is not relevant to the choice of reflections for refinement. *R*-factors based on *F*^2^ are statistically about twice as large as those based on *F*, and *R*- factors based on ALL data will be even larger.

### Fractional atomic coordinates and isotropic or equivalent isotropic displacement parameters (Å^2^)

**Table:**

	*x*	*y*	*z*	*U*_iso_*/*U*_eq_	
N2	0.34781 (11)	0.8437 (3)	0.1581 (3)	0.0417 (7)	
C18	0.33011 (15)	0.6798 (3)	−0.0211 (4)	0.0400 (8)	
C6	0.48920 (14)	0.7455 (3)	0.3722 (4)	0.0392 (8)	
C7	0.49683 (14)	0.8475 (3)	0.2692 (4)	0.0380 (8)	
C5	0.53459 (15)	0.7161 (3)	0.4735 (4)	0.0451 (9)	
C17	0.36872 (14)	0.7631 (4)	0.0641 (4)	0.0410 (9)	
C21	0.25650 (16)	0.5236 (3)	−0.1831 (4)	0.0437 (9)	
C9	0.59566 (15)	0.8817 (4)	0.3653 (4)	0.0461 (9)	
O1	0.16551 (13)	0.4657 (3)	−0.2656 (4)	0.0673 (9)	
C8	0.55013 (14)	0.9175 (3)	0.2655 (4)	0.0394 (9)	
C15	0.44934 (13)	0.8788 (3)	0.1582 (4)	0.0408 (8)	
H15A	0.4619	0.9549	0.1013	0.049*	
N3	0.39237 (12)	0.9097 (3)	0.2234 (4)	0.0434 (7)	
C29	0.42219 (16)	1.1131 (3)	0.3361 (4)	0.0437 (9)	
H29A	0.4595	1.1022	0.2991	0.052*	
C1	0.43743 (16)	0.6694 (3)	0.3833 (4)	0.0464 (9)	
H1A	0.4074	0.6842	0.3178	0.056*	
C27	0.35497 (16)	1.2377 (4)	0.4779 (5)	0.0594 (11)	
H27A	0.3468	1.3096	0.5367	0.071*	
C11	0.65023 (17)	0.9509 (4)	0.3584 (5)	0.0613 (12)	
H11A	0.6801	0.9270	0.4211	0.074*	
C14	0.56258 (15)	1.0242 (3)	0.1698 (5)	0.0482 (9)	
H14A	0.5339	1.0515	0.1053	0.058*	
C24	0.37940 (15)	1.0216 (3)	0.3050 (4)	0.0409 (9)	
C10	0.58675 (16)	0.7836 (4)	0.4662 (4)	0.0506 (10)	
H10A	0.6164	0.7624	0.5307	0.061*	
N1	0.21739 (15)	0.4423 (3)	−0.2714 (4)	0.0558 (9)	
C23	0.35171 (16)	0.5779 (4)	−0.1053 (5)	0.0546 (11)	
H23A	0.3915	0.5628	−0.1076	0.066*	
C2	0.43091 (17)	0.5761 (4)	0.4868 (5)	0.0564 (11)	
H2A	0.3967	0.5277	0.4904	0.068*	
C20	0.23398 (15)	0.6237 (4)	−0.1030 (4)	0.0476 (10)	
H20A	0.1942	0.6399	−0.1041	0.057*	
O2	0.23825 (14)	0.3550 (3)	−0.3453 (4)	0.0887 (11)	
C28	0.40961 (17)	1.2200 (4)	0.4218 (5)	0.0509 (10)	
H28A	0.4385	1.2810	0.4419	0.061*	
C12	0.65894 (18)	1.0493 (4)	0.2635 (6)	0.0689 (13)	
H12A	0.6946	1.0921	0.2605	0.083*	
C16	0.43391 (13)	0.7676 (4)	0.0517 (4)	0.0453 (9)	
H16A	0.4461	0.7885	−0.0466	0.054*	
H16B	0.4514	0.6852	0.0810	0.054*	
C13	0.61422 (17)	1.0873 (4)	0.1690 (5)	0.0577 (11)	
H13A	0.6201	1.1567	0.1051	0.069*	
C3	0.47497 (19)	0.5511 (4)	0.5891 (5)	0.0590 (11)	
H3A	0.4693	0.4889	0.6617	0.071*	
C25	0.32422 (17)	1.0409 (4)	0.3603 (5)	0.0566 (11)	
H25A	0.2949	0.9812	0.3393	0.068*	
C19	0.27035 (15)	0.7011 (3)	−0.0203 (4)	0.0474 (9)	
H19A	0.2549	0.7680	0.0365	0.057*	
C4	0.52555 (19)	0.6179 (4)	0.5811 (5)	0.0584 (11)	
H4A	0.5550	0.5993	0.6471	0.070*	
C22	0.31528 (17)	0.4985 (4)	−0.1856 (5)	0.0572 (11)	
H22A	0.3301	0.4294	−0.2403	0.069*	
C26	0.31263 (17)	1.1481 (4)	0.4460 (6)	0.0679 (13)	
H26A	0.2754	1.1600	0.4829	0.082*	

### Atomic displacement parameters (Å^2^)

**Table:**

	*U*^11^	*U*^22^	*U*^33^	*U*^12^	*U*^13^	*U*^23^
N2	0.0337 (16)	0.0427 (16)	0.0487 (18)	0.0034 (14)	−0.0089 (15)	−0.0072 (16)
C18	0.0364 (18)	0.0455 (19)	0.038 (2)	−0.0010 (16)	−0.0049 (17)	−0.0013 (18)
C6	0.0333 (19)	0.0358 (18)	0.048 (2)	0.0078 (15)	−0.0037 (16)	−0.0067 (18)
C7	0.0317 (18)	0.0382 (18)	0.044 (2)	0.0055 (15)	−0.0054 (16)	−0.0067 (18)
C5	0.046 (2)	0.044 (2)	0.045 (2)	0.0123 (17)	−0.0091 (19)	−0.005 (2)
C17	0.0322 (18)	0.049 (2)	0.041 (2)	0.0033 (16)	−0.0077 (16)	−0.0047 (19)
C21	0.040 (2)	0.052 (2)	0.0393 (19)	−0.0063 (18)	−0.0050 (18)	−0.0046 (19)
C9	0.032 (2)	0.054 (2)	0.052 (2)	0.0026 (17)	−0.0061 (17)	−0.014 (2)
O1	0.0474 (17)	0.081 (2)	0.074 (2)	−0.0142 (15)	−0.0129 (17)	−0.0069 (18)
C8	0.0347 (19)	0.042 (2)	0.042 (2)	0.0047 (15)	−0.0029 (16)	−0.0111 (18)
C15	0.0331 (18)	0.044 (2)	0.045 (2)	0.0027 (15)	−0.0080 (17)	−0.0037 (19)
N3	0.0310 (15)	0.0459 (17)	0.0534 (18)	0.0040 (14)	−0.0084 (15)	−0.0110 (16)
C29	0.041 (2)	0.041 (2)	0.049 (2)	0.0030 (17)	−0.0015 (18)	−0.0050 (19)
C1	0.041 (2)	0.042 (2)	0.056 (2)	0.0045 (17)	−0.0084 (19)	−0.001 (2)
C27	0.055 (3)	0.052 (2)	0.071 (3)	0.002 (2)	0.005 (2)	−0.022 (2)
C11	0.038 (2)	0.074 (3)	0.072 (3)	0.002 (2)	−0.012 (2)	−0.015 (3)
C14	0.040 (2)	0.050 (2)	0.055 (2)	0.0007 (18)	0.0018 (19)	−0.004 (2)
C24	0.040 (2)	0.0387 (19)	0.044 (2)	0.0035 (16)	−0.0075 (17)	−0.0030 (18)
C10	0.038 (2)	0.060 (2)	0.054 (3)	0.0114 (19)	−0.014 (2)	−0.012 (2)
N1	0.054 (2)	0.064 (2)	0.050 (2)	−0.0117 (18)	−0.0066 (18)	−0.003 (2)
C23	0.035 (2)	0.076 (3)	0.053 (2)	0.0052 (19)	−0.0053 (19)	−0.021 (2)
C2	0.054 (2)	0.045 (2)	0.071 (3)	0.0012 (19)	0.001 (2)	0.003 (2)
C20	0.0313 (19)	0.055 (2)	0.056 (2)	0.0010 (17)	−0.0054 (19)	−0.002 (2)
O2	0.075 (2)	0.097 (2)	0.094 (3)	−0.007 (2)	−0.001 (2)	−0.055 (2)
C28	0.048 (2)	0.041 (2)	0.064 (3)	0.0001 (18)	−0.004 (2)	−0.005 (2)
C12	0.048 (3)	0.071 (3)	0.088 (4)	−0.017 (2)	0.002 (3)	−0.012 (3)
C16	0.0333 (19)	0.059 (2)	0.044 (2)	0.0019 (17)	−0.0053 (17)	−0.010 (2)
C13	0.046 (2)	0.060 (3)	0.067 (3)	−0.009 (2)	0.005 (2)	−0.007 (2)
C3	0.071 (3)	0.047 (2)	0.059 (3)	0.013 (2)	0.001 (2)	0.011 (2)
C25	0.039 (2)	0.056 (2)	0.074 (3)	−0.0022 (18)	0.004 (2)	−0.019 (2)
C19	0.040 (2)	0.047 (2)	0.055 (2)	0.0028 (17)	−0.0064 (19)	−0.012 (2)
C4	0.061 (3)	0.056 (2)	0.058 (3)	0.018 (2)	−0.011 (2)	0.005 (2)
C22	0.048 (2)	0.070 (3)	0.053 (2)	0.006 (2)	−0.002 (2)	−0.023 (2)
C26	0.044 (2)	0.073 (3)	0.087 (4)	0.005 (2)	0.013 (2)	−0.029 (3)

### Geometric parameters (Å, °)

**Table:**

N2—C17	1.287 (4)	C27—C26	1.368 (5)
N2—N3	1.366 (4)	C27—C28	1.372 (5)
C18—C23	1.388 (5)	C27—H27A	0.9300
C18—C19	1.393 (5)	C11—C12	1.345 (6)
C18—C17	1.458 (5)	C11—H11A	0.9300
C6—C7	1.417 (5)	C14—C13	1.352 (5)
C6—C1	1.426 (5)	C14—H14A	0.9300
C6—C5	1.432 (5)	C24—C25	1.382 (5)
C7—C8	1.420 (5)	C10—H10A	0.9300
C7—C15	1.530 (5)	N1—O2	1.219 (4)
C5—C10	1.386 (5)	C23—C22	1.380 (5)
C5—C4	1.423 (6)	C23—H23A	0.9300
C17—C16	1.506 (5)	C2—C3	1.407 (6)
C21—C20	1.361 (5)	C2—H2A	0.9300
C21—C22	1.378 (5)	C20—C19	1.379 (5)
C21—N1	1.469 (5)	C20—H20A	0.9300
C9—C10	1.381 (6)	C28—H28A	0.9300
C9—C8	1.440 (5)	C12—C13	1.402 (6)
C9—C11	1.442 (5)	C12—H12A	0.9300
O1—N1	1.219 (4)	C16—H16A	0.9700
C8—C14	1.429 (5)	C16—H16B	0.9700
C15—N3	1.476 (4)	C13—H13A	0.9300
C15—C16	1.540 (5)	C3—C4	1.351 (6)
C15—H15A	0.9800	C3—H3A	0.9300
N3—C24	1.398 (4)	C25—C26	1.374 (5)
C29—C28	1.375 (5)	C25—H25A	0.9300
C29—C24	1.387 (5)	C19—H19A	0.9300
C29—H29A	0.9300	C4—H4A	0.9300
C1—C2	1.354 (5)	C22—H22A	0.9300
C1—H1A	0.9300	C26—H26A	0.9300
			
C17—N2—N3	109.2 (3)	C25—C24—N3	120.7 (3)
C23—C18—C19	118.3 (3)	C29—C24—N3	120.5 (3)
C23—C18—C17	121.2 (3)	C9—C10—C5	121.4 (3)
C19—C18—C17	120.6 (3)	C9—C10—H10A	119.3
C7—C6—C1	123.4 (3)	C5—C10—H10A	119.3
C7—C6—C5	119.9 (3)	O2—N1—O1	123.6 (3)
C1—C6—C5	116.7 (3)	O2—N1—C21	118.6 (3)
C6—C7—C8	119.5 (3)	O1—N1—C21	117.8 (3)
C6—C7—C15	120.7 (3)	C22—C23—C18	121.3 (3)
C8—C7—C15	119.8 (3)	C22—C23—H23A	119.3
C10—C5—C4	120.7 (4)	C18—C23—H23A	119.3
C10—C5—C6	119.8 (4)	C1—C2—C3	121.2 (4)
C4—C5—C6	119.6 (3)	C1—C2—H2A	119.4
N2—C17—C18	120.4 (3)	C3—C2—H2A	119.4
N2—C17—C16	113.8 (3)	C21—C20—C19	119.7 (3)
C18—C17—C16	125.8 (3)	C21—C20—H20A	120.1
C20—C21—C22	121.5 (3)	C19—C20—H20A	120.1
C20—C21—N1	119.3 (3)	C27—C28—C29	120.9 (4)
C22—C21—N1	119.2 (3)	C27—C28—H28A	119.6
C10—C9—C8	120.3 (3)	C29—C28—H28A	119.6
C10—C9—C11	120.9 (4)	C11—C12—C13	120.0 (4)
C8—C9—C11	118.8 (4)	C11—C12—H12A	120.0
C7—C8—C14	124.8 (3)	C13—C12—H12A	120.0
C7—C8—C9	119.1 (3)	C17—C16—C15	101.8 (3)
C14—C8—C9	116.1 (3)	C17—C16—H16A	111.4
N3—C15—C7	114.1 (3)	C15—C16—H16A	111.4
N3—C15—C16	102.2 (3)	C17—C16—H16B	111.4
C7—C15—C16	115.9 (3)	C15—C16—H16B	111.4
N3—C15—H15A	108.1	H16A—C16—H16B	109.3
C7—C15—H15A	108.1	C14—C13—C12	120.7 (4)
C16—C15—H15A	108.1	C14—C13—H13A	119.6
N2—N3—C24	118.5 (3)	C12—C13—H13A	119.6
N2—N3—C15	112.5 (3)	C4—C3—C2	119.6 (4)
C24—N3—C15	125.6 (3)	C4—C3—H3A	120.2
C28—C29—C24	120.1 (3)	C2—C3—H3A	120.2
C28—C29—H29A	120.0	C26—C25—C24	120.2 (4)
C24—C29—H29A	120.0	C26—C25—H25A	119.9
C2—C1—C6	121.7 (3)	C24—C25—H25A	119.9
C2—C1—H1A	119.1	C20—C19—C18	120.5 (3)
C6—C1—H1A	119.1	C20—C19—H19A	119.7
C26—C27—C28	119.0 (4)	C18—C19—H19A	119.7
C26—C27—H27A	120.5	C3—C4—C5	121.2 (4)
C28—C27—H27A	120.5	C3—C4—H4A	119.4
C12—C11—C9	121.5 (4)	C5—C4—H4A	119.4
C12—C11—H11A	119.2	C21—C22—C23	118.6 (4)
C9—C11—H11A	119.2	C21—C22—H22A	120.7
C13—C14—C8	122.8 (4)	C23—C22—H22A	120.7
C13—C14—H14A	118.6	C27—C26—C25	121.1 (4)
C8—C14—H14A	118.6	C27—C26—H26A	119.5
C25—C24—C29	118.8 (3)	C25—C26—H26A	119.5

### Hydrogen-bond geometry (Å, °)

**Table:**

*D*—H···*A*	*D*—H	H···*A*	*D*···*A*	*D*—H···*A*
C1—H1A···N2	0.93	2.59	3.422 (5)	150

## References

[bb1] Christoph, J. F., Liuchun, Y. & Donald, G. V. (2003). *J. Am. Chem. Soc* **125**, 3799–3812.

[bb2] Krishna, R., Velmurugan, D., Murugesan, R., Shanmuga Sundaram, M. & Raghunathan, R. (1999). *Acta Cryst.* C**55**, 1676–1677.

[bb3] Parmar, S. S., Pandey, B. R., Dwivedi, C. & Harbinson, R. D. (1974). *J. Pharm. Sci.* **63**, 1152–1155. 10.1002/jps.2600630730 4850598

[bb4] Prasad, Y. R., Rao, A. L., Prasoona, K., Murali, K. & Kumar, P. R. (2005). *Bioorg. Med. Chem. Lett.* **15**, 5030–5034. 10.1016/j.bmcl.2005.08.040 16168645

[bb5] Rigaku (2005). *CrystalClear* Rigaku Corporation, Tokyo, Japan.

[bb6] Shaharyar, M., Siddiqui, A. A. & Ali, M. A. (2006). *Bioorg. Med. Chem. Lett.* **16**, 4571–4574. 10.1016/j.bmcl.2006.06.021 16784842

[bb7] Sheldrick, G. M. (2008). *Acta Cryst.* A**64**, 112–122. 10.1107/S0108767307043930 18156677

